# Clinical significance of serum CA125 in diffuse malignant mesothelioma

**DOI:** 10.1186/s40064-016-1998-7

**Published:** 2016-03-24

**Authors:** Xu Cheng, Hong-feng Gou, Ji-yan Liu, De-yun Luo, Meng Qiu

**Affiliations:** Department of Medical Oncology, Cancer Center, West China Hospital, Sichuan University, No. 37, Guoxue Xiang Street, Chengdu, 610041 Sichuan Province China; West China School of Medicine, Sichuan University, Chengdu, Sichuan China

**Keywords:** CA-125 antigen, Drug therapy, Mesothelioma, Prognosis

## Abstract

**Background:**

Malignant mesothelioma (MM) is a rare and fatal neoplasm. For diffuse malignant mesothelioma (DMM) patients that were not suitable for cytoreductive surgery and hyperthermic intraperitoneal chemotherapy, systemic chemotherapy is the main treatment. There are no convenient tumor markers to predict the efficacy of treatment and disease progression. This study aimed to evaluate serum CA125 level as a biochemical marker of response to therapy and prognosis in patients with DMM.

**Methods:**

A retrospective study was performed in a single medical institution from April 2008 to April 2014. Overall survival (OS) and prognostic factors were assessed.

**Results:**

Forty-one patients were included with a median age of 53 years. The median OS of all patients was 10 months. Patients with baseline CA125 > 280 U/ml had worse OS compared with the patients that baseline CA125 ≤ 280 U/ml. Baseline level of CA125, stage of disease, primary tumor location and systemic chemotherapy were independent prognostic factors associated with OS. In patients who received systemic chemotherapy, the decline in serum CA125 was associated with favorable OS and objective response according to modified Response Evaluation Criteria in Solid Tumors criteria.

**Conclusions:**

The baseline level of serum CA125, accompanied with stage of disease, primary tumor location and systemic chemotherapy, could be regarded as independent prognostic factors for DMM patients. Otherwise, the change in serum CA125 can predict OS and response to systemic chemotherapy.

## Background

Malignant mesothelioma (MM) is a rare and fatal mesenchymal neoplasm arising from the serosal cavities (Kebapci et al. 2003), commonly in pleura and peritoneum, with a natural median survival of 6 months (Ruffie 1991; de Pangher Manzini et al. 1993). Treatment strategy for MM including palliative cytoreductive surgery (CRS), systemic chemotherapy and intrapeural or intraperitoneal chemotherapy does not obtain satisfactory results (Nonaka et al. 2005; Hadi et al. 2006; Herndon et al. 1998; Vogelzang et al. 2003; Mineo and Ambrogi 2012), with the median survival of less than 12 months (Creaney et al. 2013; Robinson and Lake 2005; Robinson et al. 2003; Zucali et al. 2011). Published paper demonstrated that cytoreductive surgery and hyperthermic intraperitoneal chemotherapy (CRS/HIPEC) is a potentially curative treatment for malignant peritoneal mesothelioma (MPeM) (Alexander et al. 2013).But because of the difficulty in early diagnosing, most patients are initially diagnosed in advanced stage that is not candidate for curative surgery (Grigoriu et al. 2007; Kao et al. 2011).

The serological biomarkers for response assessment and prognosis of diffuse malignant mesothelioma (DMM) are very useful and important for therapeutic decision-making. However, there is still no accepted tumor marker in routine clinical practice. Previous studies demonstrated that soluble mesothelin, with a sensitivity of 50 % and a specificity of 95 % (Robinson et al. 2003; Creaney et al. 2007), could be a relevant tumor marker and the baseline serum level of mesothelin is an independent predictor of overall survival (Hollevoet et al. 2011; Linch et al. 2014). But few Chinese medical institutions had carried out mesothelin concentration. In clinical practice, we found that patients with DMM usually have elevated level of serum CA125. Furthermore, the elevated level of serum CA125 would decline when patients received systemic chemotherapy. Recent studies demonstrated mesothelin-CA125 binding is high affinity and co-expressed in mesothelioma (Rump et al. 2004; Gubbels et al. 2006; Kaneko et al. 2009). These evidence suggested elevated levels of serum CA125 might be sensitive for predicting disease progression (Kebapci et al. 2003; Hedman et al. 2003; Baratti et al. 2009), implying serum CA125 could be a potential prognostic marker. Similarly, there is no certain tumor marker for efficacy of systemic chemotherapy in DMM patients.

CA125 is a high molecular weight transmembrane glycoprotein well known as an independent predictor of progression-free survival (PFS) and overall survival (OS) in epithelial ovarian cancer (Chen et al. 2013). Furthermore, CA125 test is a regular and convenient tumor marker in clinical practice in China.

This retrospective study aimed to evaluate the prognostic and predictive value of the baseline levels of serum CA125 and change in serum CA125 during treatment in patients with DMM.

## Methods

### Patients

We identified patients attending Cancer Center of West China Hospital from April 2008 to April 2014 with a histologically or cytologically confirmed diagnosis of MM. All included patients were at stage III or IV that were unsuitable to curative surgery (Edge et al. 2010; Troshichev et al. 1988). The eligibility criteria included that age ≥18 years; Eastern Cooperative Oncology Group (ECOG) performance status was ≤2; histologically or cytologically confirmed diagnosis of MM and immunohistochemical tests excluded the possibilities of others metastatic carcinomas; must test at least the baseline level of serum CA125 before treatment and had follow-up data in detail. Patients with history of other concomitant neoplasm and chronic organ disorders that may affect the levels of serum CA125 were excluded. This study was approved by the Medical Ethical Committee of West China Hospital of Sichuan University (Chengdu, China) and written informed consent for data collection has been obtained.

In subgroup analysis to evaluate the change in serum CA125 as the indicator for the efficacy of systemic chemotherapy, we selected candidates from overall patients by following inclusive criteria: received at least one cycle of systemic chemotherapy; monitored the levels of serum CA125 at least every one or two cycles of chemotherapy and had corresponding efficacy evaluated by CT or MRI according to Response Evaluation Criteria in Solid Tumors (modified RECIST) criteria.

### CA125 assay

CA125 (U/ml) levels were measured in serum using the CanAg CA125 assay (Roche Diagnostics). Assays were run according to manufacturer’s instructions. The upper limit of normal range was 35 U/ml.

### Treatment plan

All treatment strategies were selected by treating physician based on the patients’ physical and disease condition. The most common regimen of systemic chemotherapy was pemetrexed 500 mg/m^2^ and cisplatin 75 mg/m^2^ administered intravenously once every 21 days. Local chemotherapy was mostly administered as cisplatin 30–40 mg/m^2^ once every 21 days.

### Data collection

Data on age, gender, primary tumor location, histological subtype, stage of disease, ECOG performance status, surgery, systemic chemotherapy, local chemotherapy, levels of serum CA125, efficacy assessment and survival information were collected.

Normal range of serum CA125 in our hospital was 0–35 U/ml. In chemotherapy subgroup, change in serum CA125 was defined as level of serum CA125 after treatment minus the baseline level of serum CA125. Modified RECIST (Byrne and Nowak 2004) was used to evaluate the efficacy of systemic chemotherapy. OS was calculated from the date of advanced disease diagnosis until the death or the last recorded date of follow-up.

### Statistical analysis

Kaplan–Meier methods were used to conduct the best stratified level of baseline serum CA125 associated with OS and the univariate analysis of potential prognostic factors. In multivariate analysis, Cox’s proportional hazards regression model was used to find the independent prognostic predictors for patients with DMM. Wilcoxon signed-rank test and Spearman rank correlation were used to conduct the association between the changes in serum CA125 and modified RECIST assessment. A value of *P* < 0.05 were considered as statistically significant difference. The SPSS statistical software (IBM, V19) was used for all analyses.

## Results

### Patient characteristics

A total of 41 patients with DMM were included with a median age of 53 years (range from 24 to 80) and male preponderance (ratio: 2.15 to 1). The patient characteristics were shown in Table 1. Fourteen patients (34.2 %) had pathological epithelioid tumors subtype and most patients (31 patients, 75.6 %) were at stage IV. More than half of patients (23 patients, 56.1 %) had received systemic chemotherapy, whereas only 26.8 % of patients had undergone palliative CRS. The mean baseline serum CA125 was 351.3/ml (range 8.76–2017 U/ml), with the values of 31 patients (75.6 %) were >35 U/ml.

**Table 1:** Patient Characteristics

Parameters	No.	(%)
Total	41	100
Sex ratio (M:F)	28:13	
Median age, years (range)	53 (24–80)	
≤60 years	28	68.3
>60 years	13	31.7
Primary tumor location		
Pleura	23	56.1
Peritoneum	18	43.9
Histological subtype		
Epithelial	14	34.2
Sarcomatoid	1	2.4
Unspecified	26	63.4
Stage of disease		
III	10	24.4
IV	31	75.6
Performance status (ECOG)		
0–1	33	80.5
2	8	19.5
Surgery		
Cytoreductive surgery	11	26.8
non-cytoreductive surgery	30	73.2
Systemic chemotherapy		
Yes	23	56.1
No	18	43.9
Median cycles of systemic chemotherapy (range)	4 (1–15)	
Local chemotherapy		
Yes	13	31.7
Intrapleural chemotherapy	5	38.5
Intraperitoneal chemotherapy	8	61.5
No	28	68.3
Median cycles of local treatment (range)	5 (3–7)	
Baseline of serum CA125 (range)	351.3 (8.96–2017)	
≤35 U/ml	10	24.4
>35 U/ml	31	75.6

Twenty-three diffuse malignant pleural mesothelioma (DMPlM) patients and 18 diffuse malignant peritoneal mesothelioma (DMPeM) patients were included in study. In DMPeM subgroup, 8 patients underwent palliative CRS while only 3 patients received palliative cytoreduction in DMPlM subgroup. There were thirty DMM patients without any palliative surgery in our study.

### Overall survival

**Fig. 1:**
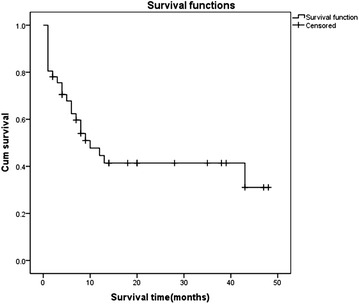
Overall survival of all 41 patients. The median overall survival (OS) for 41 patients was 10 months

**Fig. 2:**
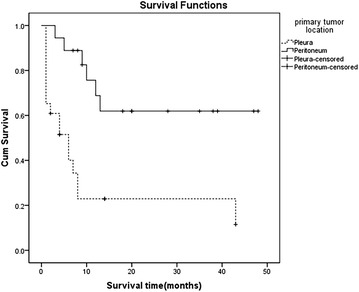
The survival of DMPlM patients and DMPeM patients subgroups. The median survival (OS) of DMPlM patients was 6 months. The median OS of DMPeM patients subgroup was longer than 24 months, *P* = 0.003

Median OS for all 41 patients was 10 months (range 1–48 months, 95 % CI 4.633–15.367, Fig. 1). OS rates at 6, 12 and 18 months was 62.4 %, 44.6 %, and 41.4 %, respectively. Eighteen patients were alive when the study finished with a median follow-up of 30 months (range 2–61 months). Survival results for DMPlM patients and DMPeM patients were presented in Fig. 2.

### Factors associated with OS in univariate analysis

**Fig. 3:**
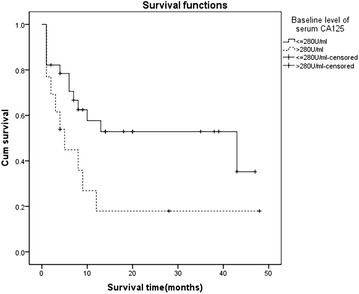
The best stratified level of baseline serum CA125 for predict DMM patients outcome. The best stratified level of baseline serum CA125 for predict DMM patients outcome was 280 U/ml (eight times of upper normal limit), *P* = 0.045

A Log-rank test was used to find out potential prognostic factors associated with OS. The best stratified level of baseline serum CA125 (shown in Table 2) associated with OS was 280 U/ml in our study (eight times of upper normal limit, *P* = 0.045, Fig. 3). Ten factors were considered into univariate analysis including age, gender, ECOG performance status, primary tumor location, pathological subtype, stage of disease, CRS, systemic chemotherapy, local chemotherapy and baseline level of serum CA125. The following factors correlated with a better outcome (shown in Table 3) included: 0–1 PS score (HR 3.013, 95 % CI 1.208–7.513, *P* = 0.018), tumor located in peritoneum (HR 0.236, 95 % CI 0.091–0.613, *P* = 0.003), epithelial subtype (HR 2.281, 95 % CI 1.261–4.124, *P* = 0.006), disease at stage III (HR 11.495, 95 % CI 1.572–87.701, *P* = 0.017), systemic chemotherapy (HR 0.340, 95 % CI 0.142–0.810, *P* = 0.015) and the baseline level of serum CA125 ≤ 280 U/ml (HR 2.214, 95 % CI 1.080–5.091, *P* = 0.045). Age, gender, CRS and local chemotherapy were not significantly related with OS.

**Table 2:** Different stratified levels of baseline serum CA125 associated with overall survival in 41 patients

Baseline of serum CA125 (U/ml)	χ^2^	*P*
≤35 versus >35	2.501	0.114
≤42.61 versus >42.61^a^	2.924	0.087
≤70 versus>70	1.715	0.190
≤138.60 versus >138.60^a^	2.339	0.126
≤140 versus >140	2.339	0.126
≤210 versus >210	1.461	0.227
≤280 versus >280	4.001	0.045
≤350 versus >350	2.227	0.136
≤489.90 versus >489.90^a^	1.352	0.245

Normal range of serum CA125 in our hospital was 0–35 U/ml

^a^The quartile of baseline serum CA125 in study was 42.61, 138.60, 489.90 U/ml, respectively

**Table 3:** Prognostic significance of clinicopatholgic variables based on univariate analysis

	No.	Median OS (months)	Survival at months (%)				Hazard ratio	P value
			6	12	18	24		
Age							1.881	0.147
≤60 years	28	13 ± 10.2	74.6	52.4	47.6	47.6		
>60 years	13	5 ± 2.2	34.6	26.0	26.0	26.0		
Gender							1.850	0.157
Male	28	43 ± 25.1	67.9	52.4	52.4	52.4		
Female	13	6 ± 2.8	45.1	22.6	11.3	11.3		
Performance status (ECOG)							3.013	0.018
0–1	33	13 ± 15.4	68.3	53.0	48.9	48.9		
2	8	2 ± 1.4	37.5	12.5	12.5	12.5		
Primary tumor location							0.236	0.003
Pleura	23	6 ± 2.1	51.5	22.9	22.9	22.9		
Pritoneum	18	–	88.9	68.8	61.9	61.9		
Histological subtype							2.281	0.006
Epithelial	14	43 ± 27.9	85.7	85.7	85.7	85.7		
Sarcomatoid	1	13	1	1	0	0		
Unspecified	26	6 ± 1.5	56.7	20.4	20.4	20.4		
Surgery							0.363	0.069
CRS	11	–	90.0	70.0	60.0	60.0		
nCRS	30	7 ± 2.0	52.1	35.8	35.8	35.8		
Systemic chemotherapy							0.340	0.015
Yes	23	43 ± 31.8	77.1	60.9	55.4	55.4		
No	18	4 ± 3.0	43.8	25.0	25.0	25.0		
Local chemotherapy							0.486	0.156
Yes	13	–	61.5	61.5	61.5	61.5		
No	28	9 ± 2.1	61.6	37.3	32.0	32.0		
Stage of disease							11.495	0.017
III	10	–	88.9	88.9	88.9	88.9		
IV	31	7 ± 2.5	52.1	31.0	26.6	26.6		
Baseline of CA125							2.214	0.045
≤280 U/ml	28	43 ± 23.4	70.6	57.7	52.9	52.9		
>280 U/ml	13	5 ± 1.7	44.9	17.9	17.9	17.9		

–, did not reach the median OS

### Factors associated with OS in multivariate analysis

Six factors were included into multivariate analysis conducted by a Cox proportional hazard model to identify the independent prognostic factors of OS. Four variables seemed to be the independent prognostic factors (shown in Table 4). Tumor located in peritoneum (HR 0.289, 95 % CI 0.096–0.870, *P* = 0.027), disease at stage III (HR 14.571, 95 % CI 1.825–116.326, *P* = 0.011), received systemic chemotherapy (HR 0.298, 95 % CI 0.099–0.900, *P* = 0.032) and baseline level of serum CA125 ≤ 280 U/ml (HR 3.412, 95 % CI 1.265–9.208, *P* = 0.015) were positive independent prognostic factors in patients with DMM.

**Table 4:** Prognostic significance of clinicopatholgic variables based on multivariate analysis

Parameters	Hazard ratio	95 % CI		*P* value
		Lower	Upper	
Tumor location (pleura vs. peritoneum)	0.289	0.096	0.870	0.027
Stage of disease (III vs. IV)	14.571	1.825	116.326	0.011
Systemic chemotherapy (no vs. yes)	0.298	0.099	0.900	0.032
Baseline level of serum CA125 (≤280 vs. >280 U/ml)	3.412	1.265	9.208	0.015

### The correlation between CA125 change and OS or radiological objective response

According the rigorous including criteria, 12 patients had regularly surveilled serum CA125 during systemic chemotherapy and finished corresponding efficacy evaluation. Wilcoxon signed-rank test assessed the differences in serum CA125 levels across the corresponding evaluation time points and found it differed significantly. Spearman rank correlation demonstrated a significant association between the change in serum CA125 and the response to systemic chemotherapy according to modified RECIST criteria (rCA125 = 0.857, *P* < 0.001, shown in Table 5).

**Table 5:** Correlation between the change in serum CA125 and the objective response

No.	Baseline CA125	CA125 after systemic CT	Change (U/ml)	Response
1	10.19	12.74	2.55	SD
2	15.10	8.62	−6.48	SD
3	17.07	42.34	25.27	PD
4	30.67	5.00	−25.67	PR
5	53.52	58.67	5.15	PD
6	75.14	16.45	−58.69	SD
7	75.94	136.90	60.96	SD
8	138.60	10.11	−128.49	SD
9	176.20	161.90	−14.30	SD
10	519.40	229.90	−289.50	SD
11	615.90	6.70	−609.20	PR
12	1918.00	462.90	−1455.10	PR

Systemic CT: systemic chemotherapy

The correlation coefficient rCA125 = 0.857; *P* < 0.001

**Fig. 4:**
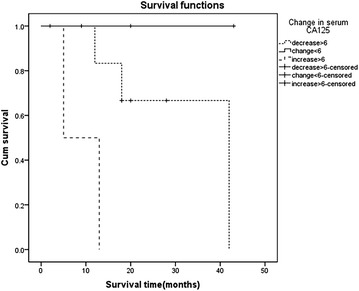
The association between change in serum CA125 and OS for patients who had received systemic chemotherapy

The median OS for patients who received systemic chemotherapy was significant longer than OS of patients without systemic chemotherapy (median survival: 43 months in chemotherapy group vs. 4 months in chemotherapy-naïve group, *P* = 0.015). Similarly, the mean level of baseline serum CA125 in systemic chemotherapy subgroup was 303.8 U/ml (range 10.19–1918 U/ml), while after receiving systemic chemotherapy, the mean level of serum CA125 was significantly declined to 96 U/ml (range 5–462.9 U/ml). Figure 4 demonstrated the association between change in serum CA125 and OS. The change was divided into three grades: a decrease >6 U/ml, a change less than 6 U/ml (stable) and an increase >6 U/ml (selected by Log-rank methods) and the decline in serum CA125 was associated with better OS in patients with systemic chemotherapy (*P* = 0.017).

## Discussion

DMM is considered as a difficult disease condition to make a diagnosis and monitor disease progression because of its unspecific clinical signs and lack of effective tumor-related markers. Level of serum CA125 was elevated in DMM patients and it co-expressed with mesothelin which was regarded as an independent prognostic factor (Hollevoet et al. 2011; Linch et al. 2014). This retrospective study aimed to gain insight in the role of serum CA125 as biomarkers of response and outcome in patients with DMM.

In our study, we found the baseline level of serum CA125 might be considered as an independent prognostic factor for patients with DMM. The serum CA125 has been shown to distinguish malignant from benign, monitor therapeutic response and prognoses the OS for pelvic and ovarian cancer (Chen et al. 2013; Meyer and Rustin 2000). And its researches for malignant mesothelioma have been reported. Hedman et al. (2003) found that the level of serum CA125 increased as mesothelioma progressed in 5 patients, while 3 patients had a stable disease with a decrease in level of CA125. Kebapci et al. (2003) found the mean baseline serum CA125 level of 11 malignant peritoneal mesothelioma (MPeM) patients was 230 U/ml. Baratti et al. (2007, 2009) reported that in non-preoperative systemic chemotherapy subgroup, diffuse malignant peritoneal mesothelioma (DMPeM) patients with baseline CA125 ≤ 35 U/ml correlated to better OS compared with patients that baseline CA125 > 35 U/ml but failed to show the association between baseline levels of CA125 and OS in entire DMPeM patients. Our study demonstrated patients with baseline CA125 > 280 U/ml (eight time of upper normal limit) had worse OS compared with the patients that baseline CA125 ≤ 280 U/ml in overall DMM patients.

Baseline level of serum CA125 ≤ 280 U/ml was also one of the independent prognostic factors for OS in multivariate analysis. Meanwhile, primary tumor location, stage of disease and systemic chemotherapy were the independent prognostic markers for DMM patients in our study and these factors have been confirmed in several papers (Vogelzang et al. 2003; Hollevoet et al. 2011; van der Bij et al. 2012; Tabata et al. 2013), supporting their close association with prognosis.

In systemic chemotherapy subgroup, the radiological responses according to modified RECIST criteria were significantly associated with relative changes in serum CA125 levels. The level of serum CA125 significantly decreased after receiving systemic chemotherapy and this tendency was accompanied with the disease control rate. Furthermore, our study demonstrated the change in serum CA125 could be a prognostic factor of OS for DMM patients who had received systemic chemotherapy. There are no previous reports focusing on this aspect.

The test of serum CA125 concentration is cheaper and more convenient adjunct to monitor patient response and prognosis than CT or MRI which are recommended for response evaluation and follow-up (Meyer and Rustin 2000; Stahel et al. 2009). Modified RECIST standard assessment is not adequate for DMM because DMM often present as a rind around the organs and pleural or peritoneal fluid rather than a spherical mass (van Klaveren et al. 2004), so we think serum CA125, like CT or MRI recommended by ESMO and NCCN guidance, should be included into regular tests to monitor the change of disease and predict the prognosis for DMM patients.

Our study had some limitations. It should be pointed out that our study was retrospective research with a small sample size. Some patients do not had specific pathological subtype may affect its role in prognosis. However, to our knowledge, our study is the first report to systematic analyzes the value of serum CA125 as a marker of response to therapy and prognosis in patients with DMM. Further validation of our findings is consequently required to elucidate the clinical value of CA125 in multicenter, larger samples prospective studies. We believe our results could provide some important information for clinical practice.

## Conclusions

In conclusion, the current study identify the best stratified level of baseline serum CA125 associated with OS was 280 U/ml. Baseline level of serum CA125, accompanied with stage of disease, primary tumor location and systemic chemotherapy, is an independent prognostic factor of DMM patients. For patients who receiving systemic chemotherapy, the change in serum CA125 could reflect the efficacy of systemic chemotherapy in keeping with modified RECIST criteria and predict OS.

## Abbreviations

MM: malignant mesothelioma
DMM: diffuse malignant mesothelioma
CRS: cytoreductive surgery
HIPEC: hyperthermic intraperitoneal chemotherapy
OS: overall survival
RECIST: Response Evaluation Criteria in Solid Tumors
MPeM: malignant peritoneal mesothelioma
PFS: progression-free survival
ECOG: Eastern Cooperative Oncology Group
DMPlM: diffuse malignant pleural mesothelioma
DMPeM: diffuse malignant peritoneal mesothelioma
ESMO: European Society for Medical Oncology
NCCN: National Comprehensive Cancer Network
